# TUSC3 inhibits cell proliferation and invasion in cervical squamous cell carcinoma via suppression of the AKT signalling pathway

**DOI:** 10.1111/jcmm.17204

**Published:** 2022-02-09

**Authors:** Fei Sun, Qiuling Jie, Qi Li, Yunjian Wei, Hong Li, Xiaojing Yue, Yanlin Ma

**Affiliations:** ^1^ Hainan Provincial Key Laboratory for Human Reproductive Medicine and Genetic Research Hainan Provincial Clinical Research Center for Thalassemia the Key Laboratory of Tropical Translational Medicine of Ministry of Education Department of Reproductive Medicine the First Affiliated Hospital of Hainan Medical University Hainan Medical University Haikou Hainan P.R. China; ^2^ Department of Obstetrics and Gynecology Nanfang Hospital Southern Medical University Guangdong China; ^3^ Haikou Key Laboratory for Preservation of Human Genetic Resource the First Affiliated Hospital of Hainan Medical University Haikou Hainan China; ^4^ Hainan Modern Women and Children’s Hospitial Reproductive Medicine Haikou Hainan China

**Keywords:** AKT signalling, invasion, prognosis, proliferation, squamous cell cervical carcinoma, TUSC3

## Abstract

The decreased expression of tumour suppressor candidate 3 (TUSC3) is associated with proliferation in several types of cancer, leading to an unfavourable prognosis. The present study aimed to assess the cellular and molecular function of TUSC3 in patients with cervical squamous cell carcinoma (CSCC). Levels of mRNA expressions of TUSC3 were analysed in CSCC tissues and six cell lines using qRT‐PCR. Immunohistochemistry(IHC) was used to evaluate the protein expression level of TUSC3 in four paired specimens, 220 paraffin‐embedded CSCC specimens and 60 cases of normal cervical tissues(NCTs), respectively. Short hairpin RNA interference was employed for TUSC3 knockdown. Cell proliferation, migration and invasion were evaluated using growth curve, MTT assay, wound healing, transwell assay and xenograft tumour model, respectively. The results demonstrated that TUSC3 mRNA and protein expression levels were downregulated in CSCC samples. Multivariate and univariate analyses indicated that TUSC3 was an independent prognostic factor for patients with CSCC. Decreased TUSC3 expression levels were significantly associated with proliferation and an aggressive phenotype of cervical cancer cells both in vitro and in vivo. Moreover, the knockdown of TUSC3 promoted migration and invasion of cancer cells, while the increased expression of TUSC3 exhibited the opposite effects. The downregulation of TUSC3 facilitated proliferation and invasion of CSCC cells through the activation of the AKT signalling pathway. Our data demonstrated that the downregulation of TUSC3 promoted CSCC cell metastasis via the AKT signalling pathway. Therefore, TUSC3 may serve as a novel prognostic marker and potential target for CSCC.

## 1 INTRODUCTION

Cervical carcinoma is the second most prevalent type of female cancer in developing countries and the main cause of death related to malignancy. Approximately 569,847 new cases and 311,365 deaths occur annually worldwide from cervical carcinoma.^1^ In recent decades, the wide implementation of the Pap smear screening program has caused a significant decrease in the incidence of cervical cancer. However, it is still a major public health problem in developing countries.^1^, ^2^ Generally, the major therapeutic modality for cervical squamous cell carcinoma (CSCC) (FIGO stage IA2–IIA) is radical hysterectomy and pelvic lymphadenectomy.^2^, ^3^, ^4^ The aggressiveness of tumour cells is closely associated with the prognosis of patients with cervical cancer.^2^, ^5^ Considerable efforts have been made to elucidate the molecular mechanisms of cell migration and invasion in tumour metastasis and specific methods have been utilized to decrease patient mortality, yet still with limitations.^5^, ^6^ Therefore, the identification of tumour‐specific markers for the diagnosis of CSCC and the evaluation of its aggressiveness are critical for high‐risk patients who require more personalized and urgent clinical intervention.

Tumour suppressor candidate 3 (TUSC3) is encoded by the TUSC3 gene, which is mapped to chromosome 8p22 and contains three prototypical different transcripts.^7^ In individual tissues and various stages of embryonic development, the expression of TUSC3 is completely different.^7^, ^8^, ^9^, ^10^, ^11^, ^12^, ^13^ More importantly, TUSC3 expression levels correlate with tumour‐suppressive or oncogenic function in specific cancer types. Previous studies have revealed that the downregulation of TUSC3 is associated with the incidence of several human cancers, including hepatocellular carcinoma,^10^ breast cancer,^11^ pancreatic cancer^12^ and ovarian cancer.^13^ Moreover, it exhibits a positive association with the proliferation, migration and invasion of tumour cells. In contrast to these observations, it was also reported that TUSC3 was overexpressed in several cancer types, such as colon cancer^14^ and non‐small cell lung cancer.^15^ Therefore, it has been suggested that TUSC3 plays intricate and important roles in different types of cancer. However, the expression pattern of TUSC3 in CSCC and its value for predicting patient survival remain unclear.

In the present study, the expression pattern of TUSC3 was investigated in patients with CSCC. The association of TUSC3 with patient prognosis was analysed, as well as its cellular and molecular function in cervical cancer cell migration and invasion.

## 2 MATERIALS AND METHODS

### 2.1 Cell culture

Hela and CaSki cells were obtained from The Cell Bank of Type Culture Collection of the Chinese Academy of Sciences. SiHa cells were purchased from China Center for Type Culture Collection. C4‐1 and HCC94 cells were purchased from OTWO (GuangZhou OTWO Biotechnology Company). The cell lines were cultured in high glucose medium DMEM (Gibco; Thermo Fisher Scientific, Inc.) supplemented with 1% non‐essential amino acids (Thermo Fisher Scientific, Inc.), 1% antibiotics (100 U/ml penicillin and 100 μg/ml streptomycin) and 10% foetal bovine serum (Gibco; Thermo Fisher Scientific, Inc.) in a 5% CO_2_ humidified atmosphere at 37°C.

### 2.2 Tissue specimens and patient information

In this retrospective study, patients were recruited from The First Affiliated Hospital of Hainan Medical University and Nanfang Hospital, Southern Medical University from 1 January 2009 to 31 December 2014. The present study was approved by the Institutional Research Ethics Committee of Nanfang Hospital and Hainan Medical University. All participants had signed written informed consent prior to the investigation. The patients who underwent chemotherapy prior to primary surgical treatment were excluded from this study. All pathological diagnoses of CSCC were confirmed by two independent pathologists. Fresh tumour specimens were timely stored in liquid nitrogen following extraction from the patients. A total of 220 paraffin‐embedded specimens were obtained from patients with CSCC and 60 NCTs from benign uterine tumour patients during hysterectomy. ANT (Adjacent Normal Tissue) was normal cervical tissue obtained from cervical cancer patient during radical hysterectomy and obtained cervical cancer tissue from the same patient at the same time. The distance between ANT and paired cancer tissue was equal or >2 cm. Patients were followed up until 31 December 2018. The detailed clinical data are summarized in Table 1.

**TABLE 1:** Clinicopathological characteristics of patients with CSCC and TUSC3 expression (*N* = 220)

Variable	N	Percentage (%)	TUSC3 expression		*p* Value
			Downregulation (*n* = 172) (%)	Normal (*n* = 48) (%)	
Age					
≥45	122	55.5	99 (45)	23 (10.5)	0.254
<45	98	44.5	73 (33.2)	25 (11.3)	
FIGO stage					
Ia2	1	0.5	0 (0)	1 (0.5)	*<0.001*
Ib1	76	34.5	42 (19)	34 (15.5)	
Ib2	59	26.8	52 (23.6)	7 (3.2)	
IIa1	22	10.0	19 (8.6)	3 (1.4)	
IIa2	33	15.0	32 (14.5)	1 (0.5)	
IIB	29	13.2	27 (12.3)	2 (0.9)	
Types of tumour growth					
Ulcerative	94	42.7	63 (28.6)	31 (14.1)	*0.002*
Endophytic	33	15.0	30 (13.6)	3 (1.4)	
Exophytic	93	42.3	79 (35.9)	14 (6.4)	
Tumour size					
≥4	110	50.0	96 (43.6)	14 (6.4)	*0.002*
<4	110	50.0	76 (34.5)	34 (15.5)	
SCC level					
≥1.5	101	45.9	85 (38.6)	16 (7.3)	0.070
<1.5	119	54.1	87 (6.4)	32 (6.4)	
Differentiation grade					
G1	9	4.1	1 (0.5)	8 (3.6)	*<0.001*
G2	77	35	58 (26.4)	19 (8.6)	
G3	134	60.9	113 (51.4)	21 (9.5)	
Stromal invasion					
≥1/2	159	72.3	131 (59.5)	28 (12.8)	*0.024*
<1/2	61	27.7	41 (18.6)	20 (9.1)	
Lymphovascular space invasion					
Yes	13	5.9	13 (5.9)	0 (0)	0.106
No	207	94.1	159 (72.3)	48 (21.8)	
Pelvic lymph node metastasis					
Yes	67	30.5	60 (27.3)	7 (3.2)	*0.007*
No	153	69.5	112 (50.9)	41 (18.6)	
Postoperative adjuvant therapy					
Yes	129	58.6	108 (49.1)	21 (9.5)	*0.028*
No	91	41.4	64 (29.1)	27 (12.3)	
Recurrence					
Yes	49	22.3	44 (20.0)	5 (2.3)	*0.030*
No	171	77.7	128 (58.2)	43 (19.5)	
Vital status at follow‐up					
Alive	172	78.2	126 (57.3)	46 (20.9)	*<0.001*
Death from	48	21.8	46 (20.9)	2 (0.9)	

Note

italic emphasis indicates statistically significant(*p < 0.05*).

### 2.3 Plasmids

The full‐length human TUSC3 gene was subcloned into the pSin‐EF1α‐puro (donation from Guangzhou Institutes of Biomedicine and Health, Chinese Academy of Sciences) lentiviral vector using the restriction enzymes *Eco*RI and *Bam*HI. Short hairpin RNAs (shRNAs) targeting TUSC3 were subcloned into the GV248‐EGFP‐puromycin (GIDE77111, GENE) lentiviral vector using the restriction enzymes *Age*I and *Eco*RI. Transfection was performed using Lipofectamine^®^ 2000 reagent (Invitrogen, Thermo Fisher Scientific, Inc.) according to the manufacturer's instructions. Following 48 h of cell culture, the cells were selected with 6 μg/ml puromycin for 7 days. The shRNA sequence used was as follows: shTUSC3 5; ‐CCTCGAAACTATTCCATGATT‐3’, which was designed according to the sequence corresponding to the GenBank accession number NM_006765.

### 2.4 Reverse transcription‐quantitative PCR (RT‐qPCR)

Total RNA was extracted from primary tumour tissues and cultured cells using TRIzol^®^ reagent (Gibco; Thermo Fisher Scientific, Inc.). cDNA was synthesized from 1 μg RNA from each sample using the iScript™ cDNA Synthesis kit (Promega Corporation) according to the manufacturer's instructions. The RT‐qPCR thermocycling conditions were the following: initial denaturation at 50°C for 2 min, 95°C for 10 min and 40 cycles of 95°C for 15 s, 56°C for 45 s. The primers for TUSC3 were as follows: Forward, 5'‐TGGATTGCTGACAGAACGGA‐3’ and reverse, 5'‐CAGAGACACCATGG CCCAAC‐3'. The primers for GAPDH were as follows: forward, 5'‐CGAGATCCC TCCAAAATCAA‐3’ and reverse, 5'‐TGTGGTCATGAGTCCTTCCA‐3'. TUSC3 expression data were normalized to GAPDH, and all experiments were performed in triplicate.

### 2.5 Cell growth curve

The cells were plated in 6‐well plates (1 × 10^5^ cells) and cultured for 6 days. The cells were enzymatic disaggregated and counted every day by using a hemocytometer for the determination of their growth curve. Each cell line experiment was performed in triplicates.

### 2.6 Western blotting

The cells were washed twice with ice‐cold PBS and lysed on ice in RIPA buffer (Cell Signaling Technology, Inc.) supplemented with a complete protease inhibitor cocktail (Roche Applied Science). Fresh tissue specimens were grounded to powder in liquid nitrogen and lysed with SDS‐PAGE sample buffer. All protein samples (30 μg) were separated with 10% SDS‐PAGE gels, transferred to PVDF membranes (Thermo Fisher Scientific, Inc.) and blocked with 5% skimmed milk in Tris‐buffered saline with 0.1% Tween‐20 (TBST) for 2 h at room temperature (RT). The membranes were incubated with anti‐TUSC3 antibody (1:500; cat. no. ab230520; Abcam) at 4°C overnight, rinsed with TBST and further incubated with goat anti‐rabbit IgG conjugated to horseradish peroxidase (Abcam) for 1 h at RT. The expression levels of TUSC3 were detected with enhanced chemiluminescence prime Western blotting detection reagents (EMD Millipore). GAPDH (1:20000; cat. no. ab8229; Abcam) was used as a loading control.

### 2.7 Immunohistochemistry (IHC)

IHC was performed as previously described.^15^, ^16^, ^17^ TUSC3 primary antibody was purchased from Abcam (cat. no. ab230520). TUSC3 staining was scored by two different pathologists who acted independently with regard to the evaluation of the intensity of staining and the proportion of positive staining. The scores were averaged. The intensity of staining was graded as follows: 3 (strong staining, ~brown), 2 (moderate staining, ~yellow brown), 1 (weak staining, ~light yellow) and 0 (no staining). The proportion of cancer cells was scored as follows: 4 (>75% positive tumour cells), 3 (51–75% positive tumour cells), 2 (26–50% positive tumour cells), 1 (6–25% positive tumour cells) and 0 (<5% positive tumour cells). The staining index for TUSC3 expression in CSCC was calculated by multiplying the two scores of the staining intensity and the proportion of positive cells. The median of all scores was used as a cut‐off value for TUSC3. An optimal cut‐off value was used as follows: a score of ≥6 was used to define tumours with high TUSC3 expression and a score of ≤4 indicated low TUSC3 expression. Positive control staining was performed in human colon cancer tissues and negative control staining was performed without primary antibody.

### 2.8 EdU assay

The EdU assay was performed as determined by the product specifications (C00031*, Guangzhou RiboBio Co., Ltd).

### 2.9 Wound healing assay

Transfected cells were grown to 80–90% confluence in 6‐well plates. The cellular layer was wounded using a sterilized tip (1 ml). Following 48 h of cell culture without serum, cell migration was monitored and microscopically photographed. The migratory ability was assessed by measuring the changes in the sizes of the wounded areas of the six fields.

### 2.10 Transwell assay

The Transwell assay (pore size 8 μm; BD Biosciences) was performed to assess the invasive ability. The membranes of the filters were coated with Matrigel (50 μl per filter; BD Biosciences). The cells were added to the upper chamber in 1% FBS medium, while the lower chamber was filled with medium containing 10% FBS. Following 24 h of cell culture, the cells on the upper surface of the membrane were removed by a cotton swab and the invaded cells on the lower membrane were stained with Giemsa. Five fields were randomly selected for cell counting on the membranes. The experiments were performed in triplicate.

### 2.11 Colony formation assay

The cells were resuspended in DMEM supplemented with 10% FBS and plated at a density of 5x10^2^ cells/well in 6‐well plates. Following 10 days of cell culture, the colonies were stained with 1% crystal violet (Sigma‐Aldrich; Merck KGaA) for 30 sec following fixation with 4% aldehyde for 5 min. The colonies were counted and photographed.

### 2.12 Tumour xenografts

All experimental procedures were approved by the Institutional Animal Care and Use Committee of Hainan Medical University. Five‐ and six‐week‐old female BALB/c nude mice (18–20 g) were purchased from the Beijing Vital River Laboratory Animal Technology Company (Beijing, CN). BALB/c nude mice were randomly divided into four groups (4 different dosages of total cells, 1 × 10^5^, 1 × 10^4^, 1 × 10^3^, 0; *n* = 5 per group). Cells with matrigel (final concentration of 25%) were inoculated into the inguinal folds subcutaneous of BALB/c nude mice. Tumour volume was calculated by the equation V = (length × width2)/2. Tumours were examined thrice weekly; length, width, and thickness measurements were obtained with callipers. On day 32, animals were euthanized, and tumours were excised and weighed.

### 2.13 Statistical analysis

Statistical analysis was performed using SPSS software (version 24.0; IBM Corp.). The association between TUSC3 levels and clinicopathological parameters was analysed by Pearson's χ^2^ or Fisher's exact tests. The analysis methods included the log‐rank test, Spearman‐rank correlation test and the Student's *t*‐test. Kaplan–Meier curves and log‐rank test were plotted to assess the effects of TUSC3 downregulation on PFS and OS. The multivariate Cox regression model was employed for analysing clinicopathological variables associated with survival. *p* < 0.05 was considered to indicate a statistically significant difference. The data are expressed as the mean ± SD of three independent experiments.

## 3 RESULTS

### 3.1 TUSC3 expression is downregulated in CSCC

**FIGURE 1:**
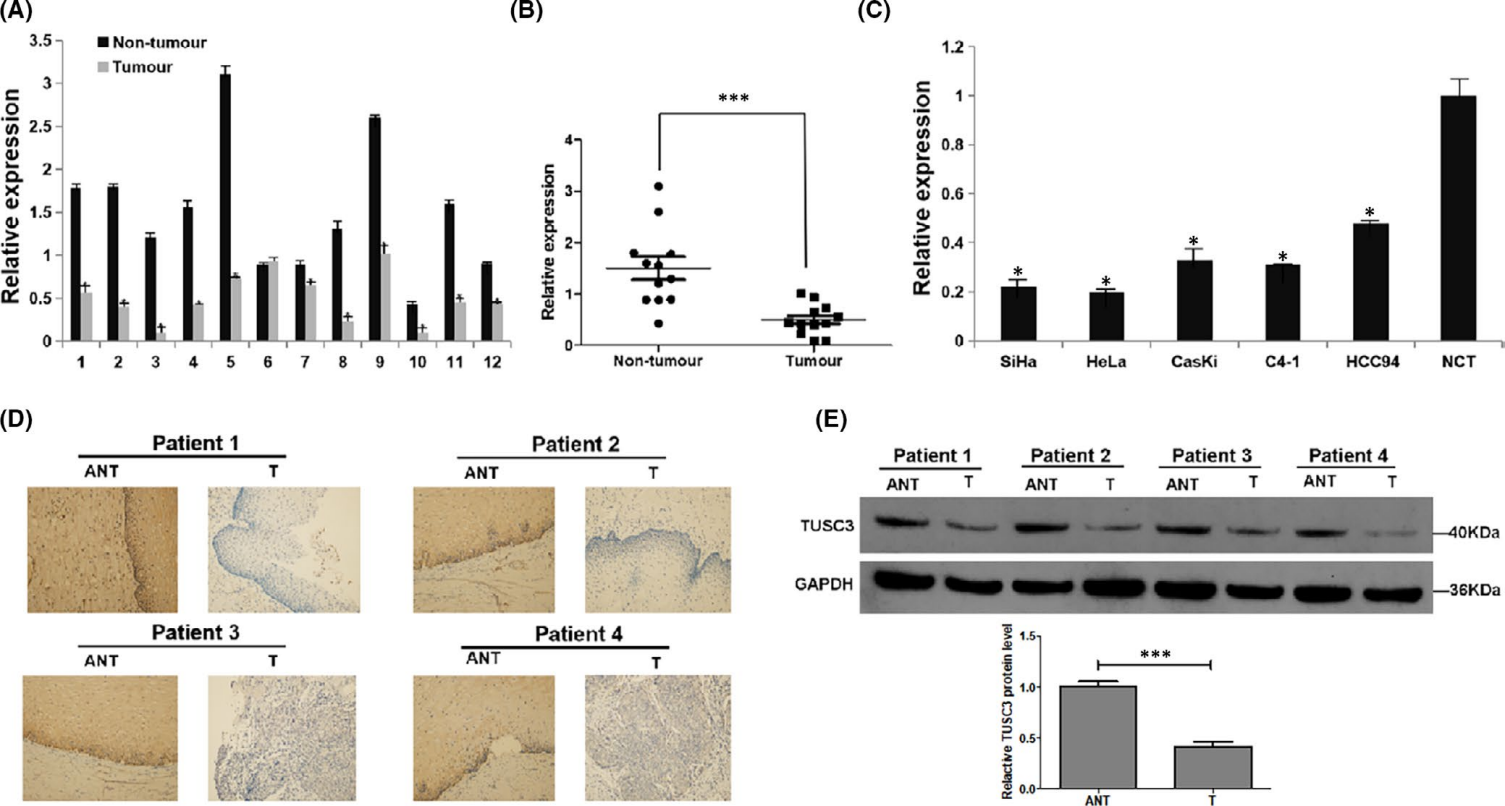
Downregulation of TUSC3 mRNA in CSCC. (A) Expression of TUSC3 mRNA in twelve paired CSCC tissues was examined by qRT‐PCR; (B) Relative expression level of TUSC3 compared with paired normal cervical tissues (NCT); *p < 0.05, ***p < 0.001. (C) Expression of TUSC3 mRNA in five cervical cancer cell lines (SiHa, HeLa, CasKi, C4‐1 and HCC94) and normal cervical tissues (NCT) was examined by qRT‐PCR. Expression levels were normalized to GAPDH. Error bars represent the means ± SD of three independent experiments. *p < 0.05. (D) Immunohistochemical assay of TUSC3 protein expression in four pairs of matched CSCC. (E) Representative images of Western blotting analyses of TUSC3 protein expression in four matched pairs of CSCC tissues (T) and adjacent noncarcinomaous tissues (ANT). GAPDH loading control. WB grey value was analysed by Image J. Error bars represent the means ± SD of three independent experiments. ***p < 0.001

The mRNA levels of TUSC3 were downregulated in both CSCC tissues and cell lines, as determined by RT‐qPCR (Figure 1A–C). Furthermore, the protein levels of TUSC3 were significantly decreased in CSCC tissues (T) compared with normal cervical tissues (NCTs), as evaluated by IHC and Western blotting analyses (Figure 1D,E).

### 3.2 TUSC3 regulates CSCC cell proliferation and invasion

**FIGURE 2:**
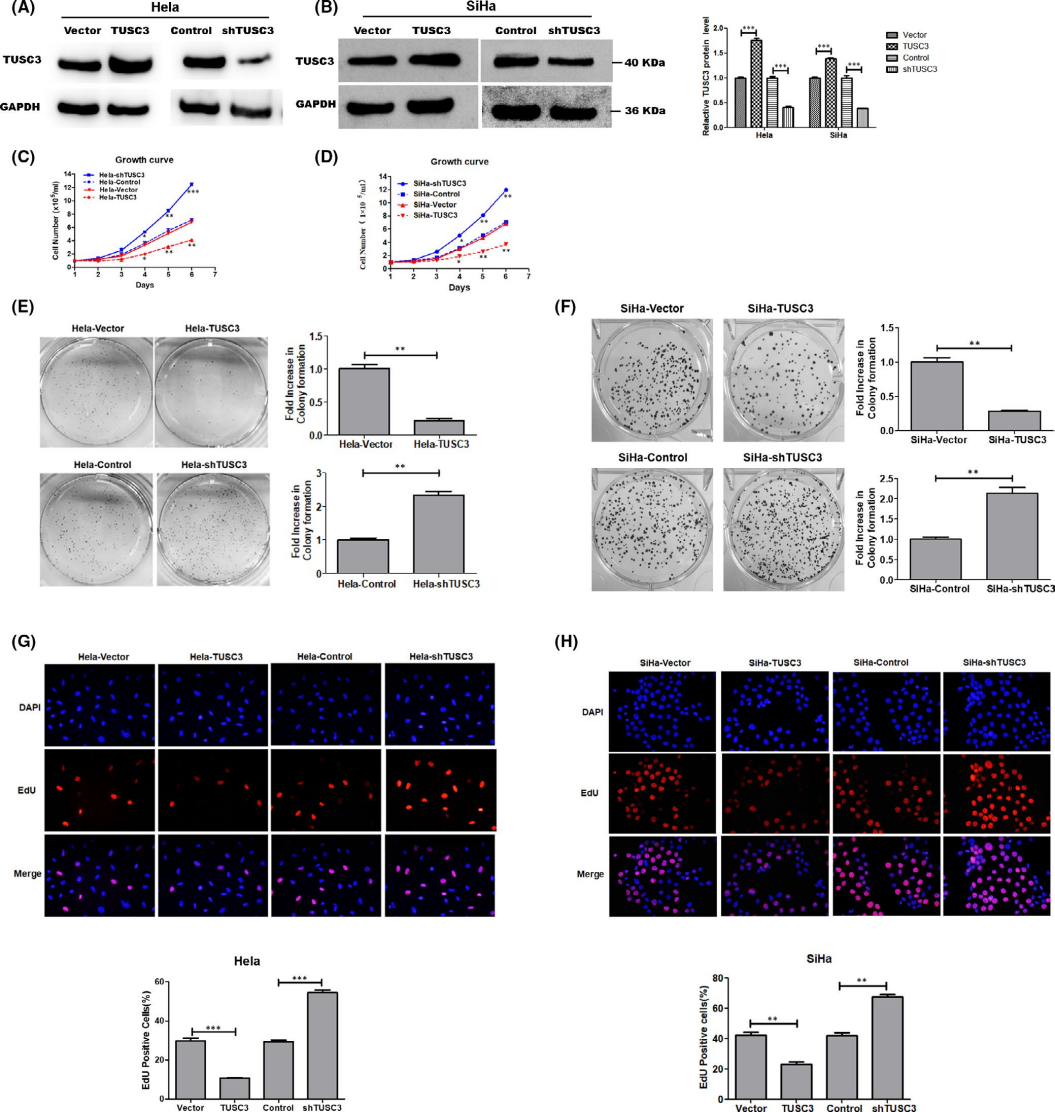
Downregulation of TUSC3 enhanced Hela and SiHa cells proliferation. (A, B) WB was performed to confirm the overexpression and knockdown of AGK in stable cell lines. GAPDH was used as an internal control. WB grey value was analysed by Image J. ***p < 0.001. (C, D) Growth curve assays in Hela and SiHa cells with TUSC3 knockdown and overexpression. Colony‐forming assay (E, F) and EdU (G, H) all indicated that downregulation of TUSC3 enhanced Hela and SiHa cells growth and overexpression of TUSC3 inhibited Hela and SiHa cells growth compared to vector control cells. Error bars represent the means ± SD of three independent experiments. *p < 0.05, **p < 0.01, ***p < 0.001

To investigate the potential function of TUSC3, stable TUSC3 overexpressing and knockdown Hela and SiHa cells (Hela/SiHa‐TUSC3 and Hela/SiHa‐shTUSC3) were established (Figure 2A,B). Hela and SiHa cells are the most usually used cervical cancer cell lines. Hela is cervical adenocarcinoma cell line and infected HPV18. SiHa is cervical squamous cell carcinoma cell line and infected HPV16.^18^ The experimental design includes the most common type of pathology and the predominant type.^19^ The growth curve assay indicated that overexpression of TUSC3 inhibited significantly the proliferation of Hela and SiHa cells (Figure 2C,D). The results from the colony‐formation and the EdU assays revealed that the downregulation of TUSC3 increased Hela and SiHa cell proliferation in vitro (Figure 2E–H).

**FIGURE 3:**
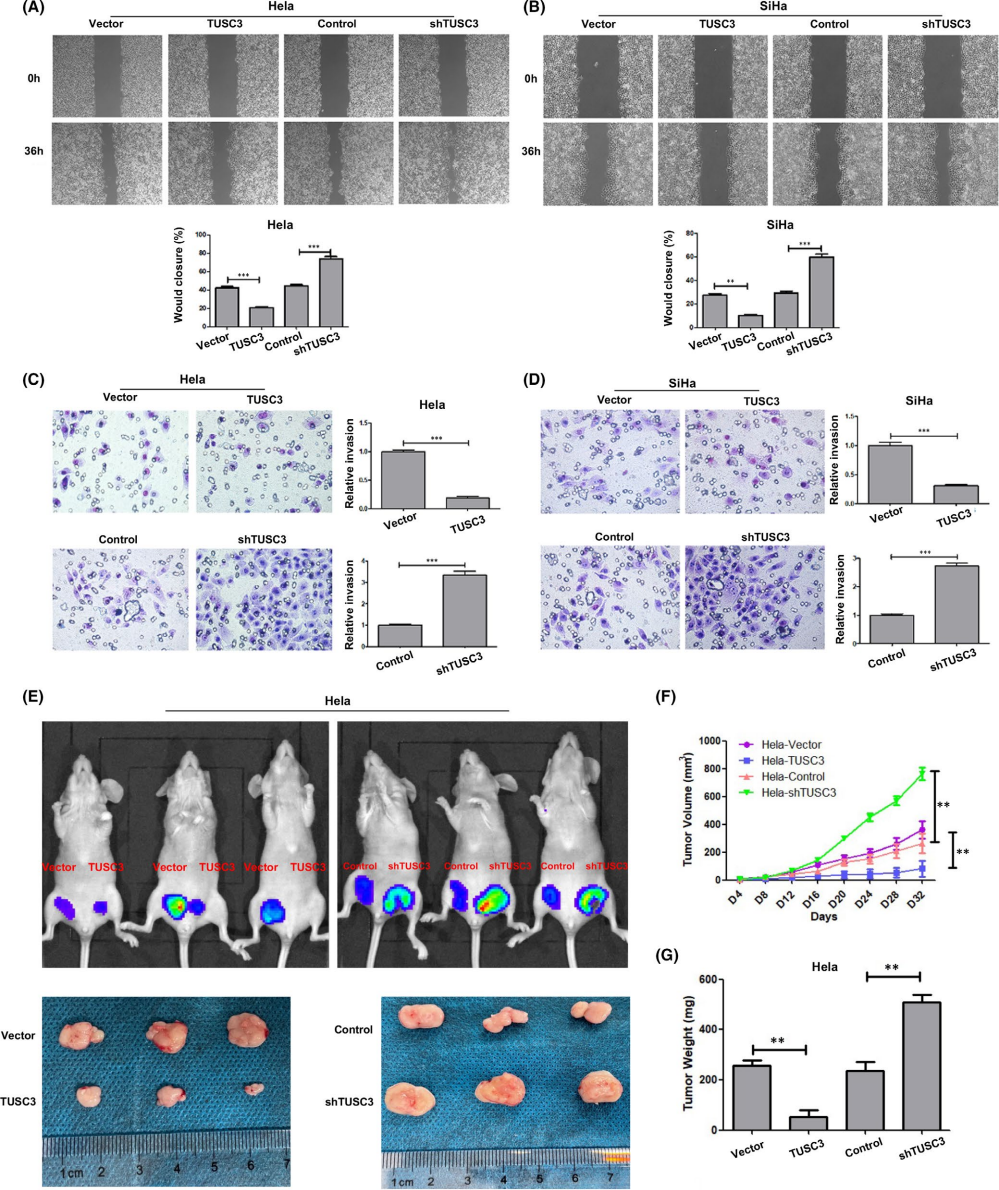
Downregulation of TUSC3 enhanced Hela and SiHa cells invasion. (A, B) Stable TUSC3 expression Hela and SiHa cells were subjected to scratch wound healing assay. The wound space was photographed at 0 and 36 h. (C, D) The invasion assays showed different cell motilities in stable Hela and SiHa cells. The depletion of TUSC3 clearly enhanced the invasion of Hela and SiHa cells. Conversely, the ectopic expression of TUSC3 inhibited the invasion of Hela and SiHa cells. (E‐G) Xenograft model in nude mice. Hela‐TUSC3, Hela‐shTUSC3 and the respective control cells were inoculated into the fat‐pad of nude mice (*n* = 5/group). (E) Representative images of tumour‐bearing mice (up panel) and images of the tumours from all mice in each group (down panel). (F) Tumour volumes were measured on the indicated days. (G) Mean tumour weights. Error bars represent the means ± SD of three independent experiments. **p < 0.01, ***p < 0.001

The wound healing assay indicated that Hela and SiHa cells with lower TUSC3 levels exhibited a significantly more extensive wound closure ability compared with that of the control sample (Figure 3A,B). The results of the Transwell assay revealed a significant increase in the invasion rate in cells with downregulated TUSC3 levels compared with the negative control group (Figure 3C,D). Therefore, it was concluded that TUSC3 may play a role in regulating CSCC cell proliferation and invasion in vitro.

We next studied the effect of TUSC3 on CSCC cells and Hela in vivo using the xenotransplantation experiment. Four different numbers (1×10^5^, 1×10^4^, 1×10^3^, 0) of TUSC3 overexpressing (Hela‐TUSC3) or knockdown (Hela‐shTUSC3) cells mixed with 25% matrigel were collected and subcutaneously inoculated into the inguinal folds of BALB/c nude mice. As shown in Figure 3E,F,G, the tumours formed by Hela‐shTUSC3 cells were significantly larger, but the tumours formed by Hela‐TUSC3 cells were significantly smaller than the tumours formed by respective control cells. Conjointly, these results demonstrate that knockdown TUSC3 enhanced the tumorigenicity of CSCC cells in vivo.

### 3.3 Downregulation of TUSC3 levels is associated with CSCC clinical features

The present study explored the expression profile of TUSC3 in 220 paraffin‐embedded archived CSCC specimens by IHC, including one stage IA, 135 stage IB, 55 stage IIA and 29 stage IIB samples. The median age was 46 years (range, 26–69 years). TUSC3 expression was downregulated in 172 (78.2%) out of 220 patients, while it was normally or overexpressed in 52 patients (52/60, 88.0%) with normal cervical tissues. Statistical analysis indicated significant associations between TUSC3 downregulation and clinicopathological characteristics of patients with CSCC, including FIGO stage (*p* < 0.001), type of tumour growth (*p* = 0.002), tumour size (*p *= 0.002), differentiation grade (*p* < 0.001), stromal invasion (*p *= 0.024) and pelvic lymph node metastasis (*p *= 0.004). TUSC3 expression was not shown to be associated with age, squamous cell carcinoma antigen (SCC) levels and other clinicopathological characteristics (Table 1).

### 3.4 Association between downregulation of TUSC3 expression and survival of patients with CSCC

**FIGURE 4:**
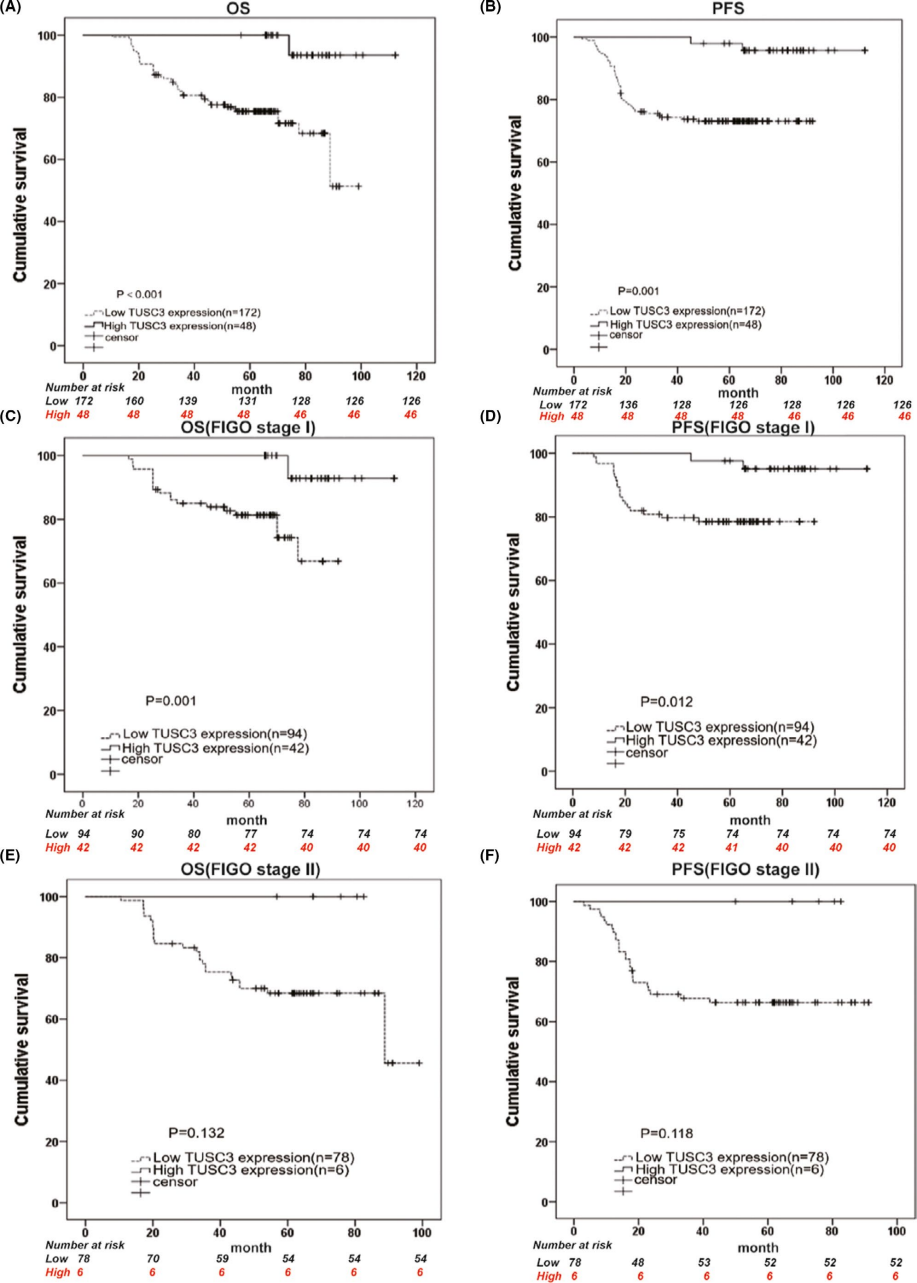
Level of TUSC3 protein expression affects the progression, free survival and overall survival of CSCC patients. Kaplan–Meier curves with univariate analysis (log‐rank) for CSCC patients with low TUSC3 expression (*n* = 172) versus high or normal TUSC3 expression (*n* = 48) for OS (A) and PFS (B). OS (C) and PFS (D) rates for cases with low TUSC3 expression versus cases with high or normal TUSC3 expression in patients with FIGO I stage. OS (E) and PFS (F) rates for cases with low TUSC3 expression versus cases with high or normal TUSC3 expression in patients with FIGO II stage

A total of 48 of 220 patients were deceased, while 172 survived, according to the last follow‐up in 2018. The 5‐year PFS and OS rates of the TUSC3 downregulation groups were 72.7% and 75.3%, respectively, whereas the rates in the normal and overexpression groups were 95.7% and 90.2%, respectively. Kaplan–Meier analysis was used to explore the association between TUSC3 downregulation and survival. A positive association was noted between the downregulation of TUSC3 expression and the concomitant OS (*p *< 0.001) and PFS (*p *= 0.001) of patients with CSCC (Figure 4A,B).

In addition, survival analysis was performed in FIGO I stage and II stage subgroups. Survival analysis revealed that TUSC3 downregulation was associated with poor OS (*p *= 0.001) and PFS (*p *= 0.012) in 136 patients with stage I (Figure 4C,D), but not with OS (*p *= 0.132) and PFS (*p *= 0.118) in 84 patients with stage II tumours (Figure 4E,F). We selected statistically significant factors from univariate analysis to include in the multivariate Cox regression analysis. Using multivariate Cox analysis, the data indicated that the downregulation of TUSC3 expression could be used as an independent prognostic factor for PFS (*p *= 0.005) and OS (*p *= 0.002) in patients with CSCC (Tables 2 and 3).

**TABLE 2:** Univariate analysis of prognostic factors for CSCC patients

Outcomes	Variable	HR	*p*	95%CI
PFS	Age	1.321	0.346	0.741–2.356
	Tumour size	2.050	*0.017*	1.134–3.705
	SCC levels	2.939	*0.001*	1.596–5.413
	Tumour differentiation	1.082	0.755	0.659–1.776
	Stromal invasion	2.441	*0.029*	1.095–5.441
	LVSI	2.565	0.031	1.089–6.042
	Lymph node metastasis	3.328	*0.001*	1.880–5.894
	FIGO stage(I vs II)	2.204	*0.006*	1.248–3.891
	Types of tumour growth	1.824	*0.002*	1.248–2.667
	TUSC3 expression	0.129	*0.005*	0.031–0.534
OS	Age	1.346	0.315	0.754–2.402
	Tumour size	2.111	*0.014*	1.167–3.820
	SCC levels	2.837	*0.001*	1.540–5.227
	Tumour differentiation	1.089	0.736	0.665–1.784
	Stromal invasion	2.369	*0.035*	1.062–5.285
	LVSI	2.515	0.064	0.949–6.204
	Lymph node metastasis	4.457	*0.035*	1.068–5.919
	FIGO stage (I vs II)	2.162	*0.008*	1.224–3.817
	Types of tumour growth	1.853	*0.002*	1.263–2.719
	TUSC3 expression	0.106	*0.002*	0.025–0.439

Note

*p < 0.05* was considered statistically significant.

Abbreviations: CI, confident interval; HR, hazard ratio; LVSI, lymphovascular space invasion; OS, overall survival; PFS, progression‐free survival.

**TABLE 3:** Multivariate analysis of prognostic factors for CSCC patients

Outcomes	Variable	HR	*p*	95%CI
PFS	Tumour size	1.364	0.333	0.728–2.556
	SCC levels	1.949	*0.040*	1.031–3.681
	Tumour differentiation	0.706	0.261	0.385–1.295
	Stromal invasion	1.965	0.102	0.874–4.415
	Lymph node metastasis	2.464	*0.003*	1.366–4.446
	FIGO stage	1.379	0.280	0.770–2.471
	Types of tumour growth	1.251	0.329	0.798–1.961
	TUSC3 expression	0.210	*0.034*	0.050–0.888
OS	Tumour size	1.393	0.307	0.737–2.630
	SCC levels	1.638	0.133	0.861–3.117
	Tumour differentiation	0.677	0.199	0.373–1.228
	Stromal invasion	1.753	0.175	0.779–3.947
	Lymph node metastasis	2.471	*0.003*	1.362–4.483
	FIGO stage	1.235	0.480	0.688–2.217
	Types of tumour growth	1.339	0.200	0.857–2.093
	TUSC3 expression	0.187	*0.023*	0.044–0.797

Note

*p < 0.05* was considered statistically significant.

Abbreviations: CI, confident interval; HR, hazard ratio; LVSI, lymphovascular space invasion; OS, overall survival; PFS, progression‐free survival.

### 3.5 TUSC3 might regulate the AKT signalling pathway in CSCC cells

**FIGURE 5:**
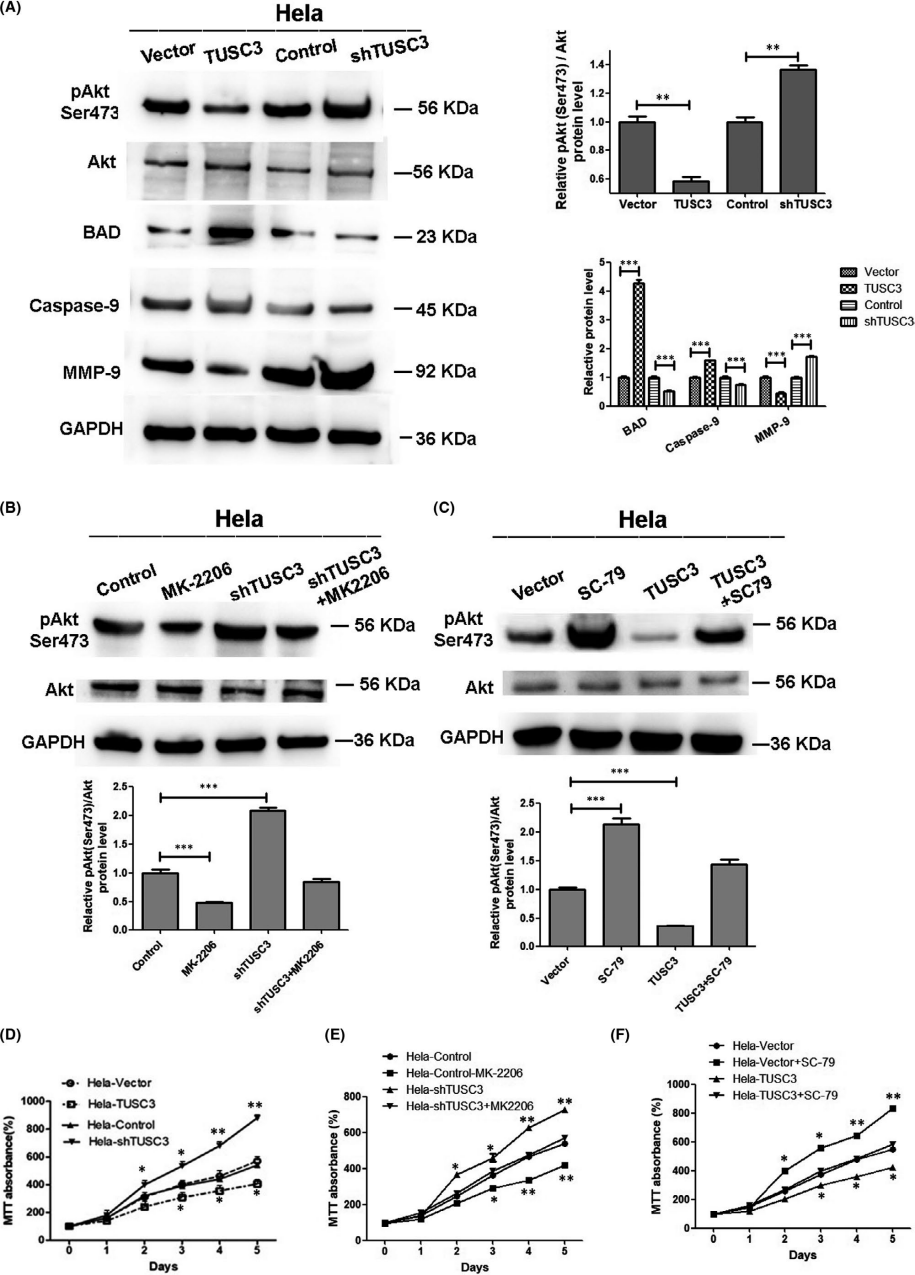
TUSC3 might regulate the AKT signalling pathway in CSCC. (A) Protein levels of total and phosphorylated Akt (Ser473), BAD, caspase‐9 and MMP9 in different groups of Hela cells were assayed by WB. GAPDH was used as the loading control. WB grey value was analysed by Image J. Error bars represent the means ± SD of three independent experiments. **p < 0.01, ***p < 0.001. WB were measured in different groups of Hela cells, in the presence or absence of 1 μM MK‐2206(B) and 4μg/ml SC‐79(C) compared to vector control cells in the absence of MK‐2206 and SC‐79. WB grey value was analysed by Image J. Error bars represent the means ± SD of three independent experiments. ***p < 0.001. (D) MTT were measured in Hela‐TUSC3, Hela‐shTUSC3 and the respective control cells. (E) MTT were measured in Hela‐TUSC3 and control cells with the AKT inhibitors MK‐2206. (E) MTT was performed to test Hela‐shTUSC3 and control cells with the AKT activator SC‐79. Error bars represent the means ± SD of three independent experiments. *p< 0.05, **p < 0.01

It was reported in previous studies that the loss of TUSC3 was associated with the activation of the AKT signalling pathway.^20^, ^21^ In order to investigate the association between TUSC3 expression and the AKT signalling pathway in CSCC, the phosphorylated levels of AKT were evaluated in HeLa cells transfected with TUSC3 plasmids. The data demonstrated that the overexpression of TUSC3 significantly inhibited AKT phosphorylation, whereas it decreased AKT activity (Figure 5A). The AKT phosphorylation site in this study is Ser473. Subsequently, the targets involved in the AKT signalling pathway were examined, including BAD, caspase‐9 and MMP9. These targets were regulated by TUSC3 in Hela cells (Figure 5A). To confirm whether TUSC3 regulates Hela cell proliferation via the AKT signalling pathway, a specific inhibitor, MK‐2206, was used to block this pathway. SC‐79, an AKT activator, was used to activate this pathway. When Hela cells were cultured in the absence of the MK‐2206 inhibitor, the downregulation of TUSC3 enhanced the expression of p‐AKT(Ser473) whereas this effect was reversed using MK‐2206 (Figure 5B). When Hela cells were cultured in the absence of the SC‐79 activator, the upregulation of TUSC3 suppressed the expression of p‐AKT(Ser473) whereas this effect was reversed using SC‐79 (Figure 5C). And the AKT inhibitors MK‐2206 could reverse the enhanced CSCC cells proliferation when TUSC3 was knockdown as shown in Figure 5D,E. The AKT activator SC‐79 could rescue the inhibited CSCC cells proliferation when TUSC3 was overexpression as shown in Figure 5F.

## 4 DISCUSSION

In the present study, the clinical significance of TUSC3 was assessed in patients with CSCC. The present study revealed that the downregulation of TUSC3 expression increased the proliferation and invasion of CSCC cells, whereas its upregulation exhibited the opposite effects. Furthermore, TUSC3 may modulate the activity of the AKT signalling pathway to promote tumour progression and metastasis. The downregulation of TUSC3 expression was associated with a high risk of metastasis and unfavourable survival in patients with CSCC. Therefore, TUSC3 may be regarded as a novel prognostic factor for patients with CSCC.

TUSC3 has been characterized as a candidate tumour suppressor gene, which acts as an important signalling hub that modulates a variety of cellular processes.^22^ Previous studies have shown that the downregulation of TUSC3 expression is tightly associated with the incidence of several human cancer types, including hepatocellular carcinoma, breast cancer, pancreatic cancer and ovarian cancer,^10^, ^11^, ^12^, ^13^ suggesting a functional association between the downregulation of TUSC3 expression and cancer progression. The present study revealed that the downregulation of TUSC3 expression may enhance aggressive clinical behaviour in CSCC and accelerate the metastatic properties of HeLa and SiHa cells, whereas the overexpression of TUSC3 exhibited the opposite effects. The analyses of lung, pancreatic, ovarian and glioblastoma cancer indicated that the risk of proliferation and invasion was higher in subjects with downregulated TUSC3 expression.^10^, ^12^, ^14^, ^21^, ^22^, ^23^, ^24^ However, the involvement of TUSC3 in CSCC, either epigenetically or gnomically, has not been previously reported. Therefore, to the best of our knowledge, the present study was the first to demonstrate the function of TUSC3 in the development of CSCC.

According to a recent study, the downregulation of TUSC3 expression may lead to lymph node or distant metastasis formation in a variety of cancer types, suggesting that the induction of cancer cell migration and invasion by TUSC3 may be the underlying pathogenic mechanism.^22^ Certain in vitro studies have suggested that the downregulation of TUSC3 may induce the proliferation and migration of ovarian cancer cells, and increase the adhesion to the extracellular matrix.^23^, ^24^ Similar results were reported in prostate cancer.^20^ The present study demonstrated that the protein and mRNA levels of TUSC3 were both downregulated in CSCC. It was further shown that the downregulation of TUSC3 expression was significantly associated with unfavourable survival outcomes and disease progression. It is important to note that a significant association between the downregulation of TUSC3 expression and the clinicopathological characteristics of patients with CSCC was observed, including FIGO stage (*p *< 0.001), types of tumour growth (*p *= 0.002), differentiation grade (*p *< 0.001), stromal invasion (*p *= 0.024), pelvic lymph node metastasis (*p *= 0.004) and recurrence (*p *= 0.03), all of which were important factors in predicting the progression and prognosis of CSCC.

The EMT (epithelial–mesenchymal transition) can change the cell epithelial cancer cells adhesion and enhance them metastasis. Primary cervical cancer with an EMT phenotype showed increased tumour progression, migration, invasion and deformation in epithelial integrity,^25^ while activation of the Akt pathway is required for MMP9‐induced EMT.^26^ It has been shown that the AKT signalling pathway exhibits a significant impact on cancer progression by regulating cell growth,^20^, ^21^, ^27^ cell apoptosis and mesenchymal transition.^10^, ^11^, ^28^, ^29^, ^30^, ^31^ It had been verified that TUSC3 could active many important pathways, including PI3K/AKT/mTOR (PI3K/AKT) and Ras‐MEK‐ERK (MAPKs), were related to the regulation of MMP9.^32^ The phosphorylate AKT could result in the overexpression of MMP9.^33^ Matrix metalloproteinase (MMP) family is an important protein family associated with the regression of the ECM (extracellular matrix) and EMT.^34^, ^35^

Previous studies have shown that silencing of TUSC3‐dependent AKT signalling in GBM cells may lead to a high level of proliferation^21^ and a similar phenomenon could be observed in prostate cancer.^20^ The relationship between AKT signalling pathway and tumour cell progression, migration, invasion in CSCC is also well‐known.^36^ Therefore, the current study investigated whether the downregulation of TUSC3 may modulate the AKT signalling pathway in CSCC cells.

Here, our analysis demonstrated an influence of TUSC3 on AKT phosphorylation and activity. The targets of the AKT signalling pathway, including BAD, caspase‐9 and MMP9 were all regulated by TUSC3. We revealed that TUSC3 may modulate the AKT and regulate MMP9 to enhance cervical cancer cells invasion. BAD and caspase‐9 are significant factors to control cell apoptosis in PI3K/AKT signal pathway. TUSC3 may regulate the AKT and influence BAD and caspase‐9 to enhance cervical cancer cells proliferation.^37^, ^38^ In the present study, application of MK‐2206 (AKT signalling inhibitor) to the cells abolished the effects of silence TUSC3 increasing the expression of p‐AKT(Ser473). Application of SC‐79 (AKT signalling activator) to the cells reversed the effects of TUSC3 decreasing the expression of p‐AKT(Ser473). And the AKT inhibitors MK‐2206 could reverse the enhanced CSCC cells proliferation when TUSC3 was knockdown as shown in Figure 5D,E. The AKT activator SC‐79 could rescue the inhibited CSCC cells proliferation when TUSC3 was overexpression as shown in Figure 5F. Therefore, knockdown TUSC3 resulted in the activated of AKT signalling pathway, which could partially explain the promotion effect on the proliferation and invasion of CSCC cells caused by this protein.

Altogether, loss of TUSC3 was accompanied by upregulation of EMT‐related pathways, decreased expression of epithelial markers together with upregulation of mesenchymal markers and resulting in enhanced cell invasion and migration.

Currently, the main therapies used for patients with CSCC include surgery followed by adjuvant therapy.^2^, ^3^, ^4^ The present study suggested that TUSC3 may act as a potential tumour suppressor gene in CSCC, which revealed a unique Janus‐like character in cancer pathogenesis and development. Notably, the role of TUSC3 was examined in specific cancer subgroups based on their FIGO stage and the prognostic potential of the downregulation of its expression was investigated in FIGO I stage subjects. This result demonstrated that TUSC3 may participate in the initial phase of CSCC carcinogenesis. For this reason, the detection of TUSC3 protein in CSCC tissues could assist in evaluating prognosis and providing guidance in the patient follow‐up schedule.

## 5 CONCLUSIONS

The present study demonstrated for the first time that the downregulation of TUSC3 was associated with the enhanced aggressiveness of CSCC cells and poor survival outcomes. This was achieved by using cell functional and survival analysis models. The downregulation of TUSC3 expression promoted CSCC cell metastasis. TUSC3 might regulate the CSCC cells through AKT signalling pathway. This feature may be used as an independent prognostic factor of CSCC patient survival. Therefore, TUSC3 may become a new prognostic biomarker for patients with CSCC.

## CONFLICT OF INTEREST

The authors declare that they have no competing interests.

## AUTHOR CONTRIBUTION

**Fei Sun:** Conceptualization (equal); Data curation (equal); Formal analysis (equal); Funding acquisition (equal); Investigation (equal); Writing – original draft (equal); Writing – review & editing (lead). **Qiuling Jie:** Methodology (equal); Resources (equal); Writing – original draft (equal). **Qi Li:** Methodology (equal); Resources (equal). **Yunjian Wei:** Investigation (equal); Methodology (equal). **Hong Li:** Resources (equal). **Xiaojing Yue:** Resources (equal). **Yanlin Ma:** Conceptualization (equal); Data curation (equal); Resources (equal); Writing – original draft (equal); Writing – review & editing (equal).

## Data Availability

The data sets used during the present study are available from the corresponding author upon reasonable request.
